# The International Medical Congress

**Published:** 1886-01

**Authors:** 


THE INTERNATIONAL MEDICAL CONGRESS.—We have repeatedly drawn our readers' attention to the progress made, both upon this side and the American side, in the direction of forming an effective arrangement for the holding of the Congress. As was pointed out, there was at one time a most hopeless state of affairs. Internal, we might almost say petty personal, interests were introduced, which were wholly foreign to a congress of scientists, which should, above everything, be cosmopolitan. The section devoted to Oral Surgery and Dentistry was, while these evil counsels prevailed, left out in the cold limbo of neglect. Dentists, we were shrewdly told, were not medical men (?) and so had no right to a place in the deliberations of those learned in the lore of medicine, or expert in the craft of surgery. However, happier and wiser counsels have now won the day, and the section in which our profession takes the most interest has been reinstated. The vast importance of the International Congresses cannot be justly appreciated unless by those who have carefully attended the meetings, and assiduously *set themselves to learn*. That America is to be the next meeting place will, unless we are much mistaken, prove of considerable importance to us dentists. The conflict between English and American dentists is still keen; we on this side have yet much to learn, and we stand an excellent chance of gaining the needed information, if we avail ourselves of this opportunity of going across the Atlantic rollers. The assumption of the Presidential chair in the section devoted to oral surgery, by Dr. Taft, is matter of congratulation. The programme of that section has not at present reached us, but we have little doubt that it will be all it should be, and will afford everyone a chance of putting on record the results of his work and experience. The rules regulating the business of the Congress are fair, and open to only friendly criticism; we append the more salient ones for the perusal of our readers.

To urge upon our English dentists "to be up and doing," will, we hope, be a work of supererogation, but it seems that as a body, they are very modest and very retiring. This is a pity. A man owes it alike to himself and his profession and his country to take some part, even if it be only that of a hearer, in all such international meetings. The Congress at

Copenhagen can hardly be considered to have been a success, so far as we are concerned. It is often and often deplored that we dentists do not get our deserts; we are ourselves to blame for this. The world is apt to accept a man at his own valuation, or a few figures below that estimate, and so if we persistently hold our tongues and show no signs of intellectual life, while America is plethoric with skilled dentists, who are likewise skilled workers, and not less important, skilled talkers, we must necessarily gain the character of being mere office drudges. The epithet will be said to show base ingratitude when there are so many leaders of men among us, who attend meetings, read papers, and teach the scientific side of our profession. It is not so, however; for if the names of the few who do thus distinguish themselves be scrutinized, they will be found to constitute a very minute minority of the possible number of men who could, if they chose, do something to forward the profession, and add to its laurels. There is yet another motive, although a sordid one, which should induce English dentists to pay more heed to our advice. The more men show the world of what material they are made, the more will the world think of them, and learn to value at its due price the services of skilled and scientific dentists.—*Editorial in British Journal of Dental Science.*

